# Serotonin Transporter Distribution in the Brainstem of Multiple-System Atrophy–Parkinsonian Type: Insights from Pathology and ^123^I-FP-CIT SPECT Findings

**DOI:** 10.2967/jnumed.124.268669

**Published:** 2026-04

**Authors:** Cong Shang, Ryunosuke Nagao, Yuichi Riku, Takashi Ichihara, Yoshitaka Inui, Masanobu Ishiguro, Yuumi Tanaka, Yasuaki Mizutani, Masanori Inoue, Yasushi Iwasaki, Mari Yoshida, Mizuki Ito, Hirohisa Watanabe, Hiroshi Toyama

**Affiliations:** 1 Department of Radiology, Fujita Health University, Toyoake, Japan;; 2 Department of Neurology, Fujita Health University, Toyoake, Japan;; 3 Institute for Medical Science of Aging, Aichi Medical University, Nagakute, Japan; and; 4 Section of Radiology, Fujita Health University Hospital, Toyoake, Japan

**Keywords:** ^123^I-FP-CIT SPECT, multiple-system atrophy, serotonin transporter, selective serotonin reuptake inhibitors, anti-SERT immunohistochemistry

## Abstract

Studies have demonstrated loss of serotonergic neurons in the brainstems of patients with multiple-system atrophy (MSA). This study aimed to semiquantitatively investigate the status of serotonin transporter (SERT) distribution in the brainstem of individuals with MSA–parkinsonian type (MSA-P) via ^123^I-2β-carbomethoxy-3β-(4-iodophenyl)nortropane (^123^I-FP-CIT) SPECT and compare it with pathologic findings in some cases. **Methods:** We administered ^123^I-FP-CIT intravenously to 19 patients with MSA-P and 17 healthy controls (HCs) and performed SPECT and MRI scans. Specific binding ratio (SBR) images were generated, and summed voxel-based SBRs for the midbrain, pons, and entire brainstem were quantified. The Mann–Whitney *U* test was used to compare the MSA-P and HC groups, and receiver operating characteristic curves were used to analyze the midbrain-to-pons ratio of the summed voxel-based SBR. Further, we assessed postmortem SERT immunohistochemistry pathology in the brainstems of representative MSA-P cases and HCs to compare the distribution and density of SERT with SPECT findings. **Results:** ^123^I-FP-CIT SPECT results revealed a significant summed voxel-based SBR decrease in the midbrain and an increase in the pons in the MSA-P group, although the brainstem summed voxel-based SBRs did not differ significantly (*P* < 0.05). The use of the midbrain-to-pons ratio for differentiation generated an area under the curve of 0.93. SERT immunostaining pathology, consistent with the ^123^I-FP-CIT SPECT findings, demonstrated a significant decrease in SERT expression in the substantia nigra and a significant increase in the pontine raphe nucleus in patients with MSA-P. **Conclusion:** Our results indicate differences in SERT distribution in the brainstems of patients with MSA-P and HCs.

## Visual Abstract

**Figure:**
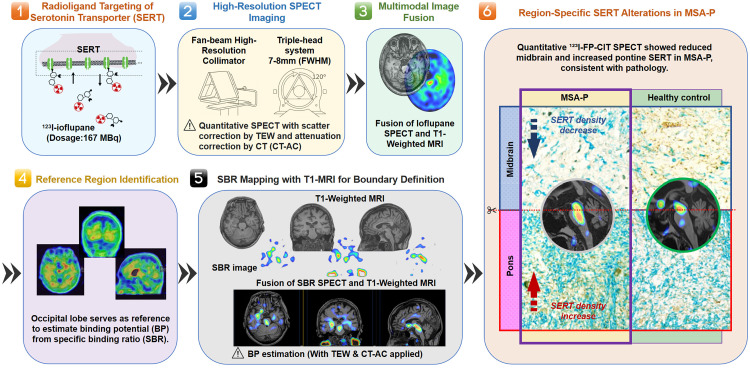

Multiple-system atrophy (MSA) is a sporadic neurodegenerative disease characterized by different degrees of parkinsonism, cerebellar ataxia, and autonomic failure throughout its progression. MSA–parkinsonian type (MSA-P) and MSA–cerebellar type are cases with predominant parkinsonism and cerebellar ataxia, respectively (1). MSA has a poor prognosis, as patients progress from disease onset to death within approximately 9 y (2).

The relatively high incidence of sudden death, which is frequently associated with respiratory complications, is an important feature of MSA (3,4). Causes of respiratory dysfunction and sudden death in patients with MSA remain unclear. Autopsy studies have indicated that significant serotonergic neuronal loss in the brainstem may cause sudden death in these patients (5–9), including those with minimal or no motor symptoms (7). Further, other studies have highlighted the importance of serotonin by demonstrating that cerebrospinal fluid levels of 5-hydroxyindoleacetic acid, a major serotonin metabolite, are significantly lower in patients with MSA than in healthy controls (HCs) (10,11)—a finding that correlates with the severity of motor symptoms, including trunk and orthostatic hypotension (11).

The invasive nature of cerebrospinal fluid testing limits its clinical use; thus, ^123^I-2β-carbomethoxy-3β-(4-iodophenyl)nortropane (^123^I-FP-CIT) SPECT provides a noninvasive alternative. Traditionally used to evaluate striatal dopamine transporters, ^123^I-FP-CIT SPECT binds to the serotonin transporter (SERT) in the brainstem, thereby enabling the visualization of serotonergic changes in vivo (12). Individuals receiving selective serotonin reuptake inhibitors (SSRIs) show a significant decrease in ^123^I-β-CIT SPECT uptake in the brainstem but not in the striatum, indicating that this decrease primarily reflects SERT binding in the brainstem (13).

SPECT is widely available and cost-effective and does not need a cyclotron despite its poorer resolution than PET. Our recent success with triple-head SPECT in obtaining ^123^I-FP-CIT SPECT data (14) using the 3-compartment model of Mintun et al. (15) to collect specific binding ratio (SBR) images has enabled semiquantitative assessments of SERT density.

In this study, we focused on the brainstem—specifically dividing our analysis into the midbrain and pons—to semiquantitatively evaluate SERT distribution in individuals with MSA-P compared with HCs. This brainstem division is particularly significant because the midbrain and pons play distinct roles in autonomic and motor control and the roles are frequently disrupted in MSA. Postmortem SERT–immunohistochemical pathology further supported the validity of our SPECT findings.

## MATERIALS AND METHODS

The Institutional Ethics Review Board of Fujita Health University (reference number HM23-296) approved this study, which adhered to the ethical standards of the 1964 Declaration of Helsinki and its subsequent amendments. The institutional review board approved both the SPECT and the pathology assessments, and all participants gave written informed consent before enrollment.

### SPECT Assessment

A cross-sectional study was conducted from September 2020 to May 2022 and included 20 patients with MSA-P and 17 HCs recruited from the Department of Neurology at Fujita Health University Hospital. We focused on MSA-P to prevent the confounding effects of pronounced brainstem atrophy observed in MSA–cerebellar type and mitigate its effects on SERT distribution interpretation.

**FIGURE 1.**
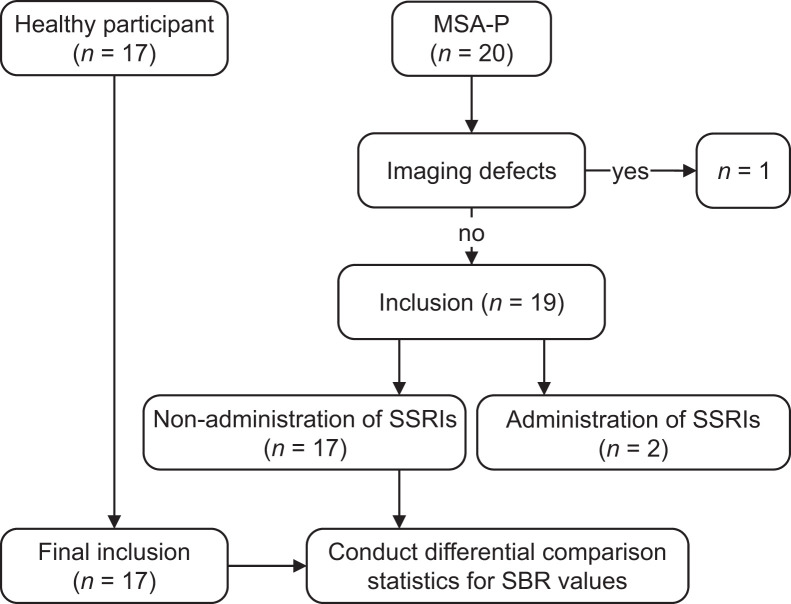
Participant inclusion flowchart for ^123^I-FP-CIT SPECT. This study included 20 patients with MSA-P and 17 HCs. One patient with MSA-P was excluded because of imaging defects. Further, 2 patients with MSA-P were excluded from group comparison statistics because they were affected by SSRI use.

Patients with MSA-P underwent initial neurologic assessment based on the criteria outlined in the Movement Disorder Society diagnostic criteria for MSA (16). One was excluded because of an MRI artifact (Fig. 1), and two were undergoing SSRI treatment. The HC group comprised 17 healthy Japanese individuals (Table 1) with no neurologic abnormalities on MRI. They scored zero on the Unified Parkinson Disease Rating Scale Part III and met additional criteria, including a Montreal Cognitive Assessment score of at least 26 and Beck Depression Inventory scores of no more than 10. Noteworthily, our previous study (14) included both MSA-P and MSA–cerebellar type, comprising 7 participants with MSA-P overlapping with the current cohort. However, that study focused on a qualitative visual assessment of peak brainstem uptake, whereas the present study used a fully quantitative, 3-dimensional (3D) region-of-interest (ROI)–based approach to assess total brainstem SBR. Thus, the 2 studies differ in terms of methodology and analytic objectives.

**TABLE 1.** Demographics and Relevant Clinical Data of Study Participants

Parameter	MSA-P	HC	*P*
^123^I-FP-CIT SPECT study			
* *Total (*n*)	19	17	
Women (*n*)	10 (61.7 y)	9 (73.3 y)	1.00*
Men (*n*)	9 (63.7 y)	8 (70.4 y)	
Mean age ± SD (y)	62.6 ± 9.4 (range, 47–78)	71.9 ± 10.3 (range, 55–99)	0.02^†^
Duration (mo)	24.8 ± 13.3	—	—
Anti-SERT immunohistochemistry study			
* *Total (*n*)	5	5	
Women (*n*)	0 (—)	0 (—)	1.00*
Men (*n*)	5 (59.4 y)	5 (59.8 y)	
Mean age ± SD (y)	59.4 ± 8.44	59.8 ± 7.53	0.83^†^
Duration (mo)	81.6 ± 56.54	—	—

* Fisher exact test.

† Mann–Whitney *U* test.

Values in parentheses following the number of women and men indicate the mean age in years.

### Equilibrium Model

**FIGURE 2.**
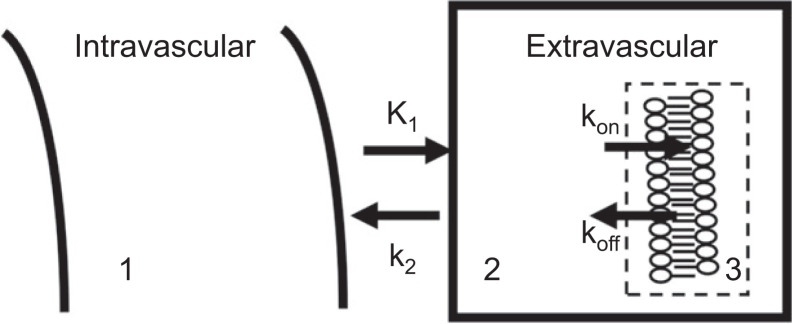
Three-compartment model was used to analyze brain tissues. Compartments 1, 2, and 3 represent possible environments for radioligand, with interactions between compartments governed by diffusion and binding kinetics (*k*_on_, *k*_off_). This figure is based on study by Mintun et al. (15).

We recently reported the association between the SBR and total SERT content in tissues (14) based on the binding potential (BP) concept proposed by Mintun et al. (15). An intravenously administered radioligand from the bloodstream (compartment 1) crosses the blood–brain barrier and reaches the brain tissue (compartment 2), where it binds to the target receptor (compartment 3) and eventually reaches an equilibrium concentration (Fig. 2) at which association and dissociation rates in compartment 3 are equal.

$${{0=k_{\mathrm{on}}f}_{2}C}_{2}\left(B_{\max}-C_{3}\right)-k_{\mathrm{off}}C_{3}.$$

Injected radioligand molar quantities were relatively low, and the radioligand concentration bound to the target (*C*_3_) was substantially lower than the target’s total binding capacity (*B*_max_). Thus, *B*_max_ − *C*_3_ would be simplified to *B*_max_. BP is defined as the ratio of *B*_max_ to *K*_D_, and *K*_D_ = *k*_on_/*k*_off_.

$$\mathrm{BP}=\frac{B_{\max}}{k_{\mathrm{off}}/k_{\mathrm{on}}}\approx \frac{C_{3}}{{f_{2}C}_{2}}.$$

The target-to-background ratio is commonly used to estimate the BP of ROI. In our study, the brainstem and occipital lobe were set as ROI and reference region, respectively. The target-to-background ratio, calculated as SBR, represents modified BP.

$$\mathrm{BP}=\frac{B_{\max}}{k_{\mathrm{off}}/k_{\mathrm{on}}}\approx \frac{C_{3}}{f_{2}C_{2}}\approx \left[{\left(\mathrm{cpm}/\mathrm{pixel}\right)}_{\mathrm{brainstem}}-{\left(\mathrm{cpm}/\mathrm{pixel}\right)}_{\mathrm{occipital}}\right]/{\left(\mathrm{cpm}/\mathrm{pixel}\right)}_{\mathrm{occipital}}.$$

Table 2 shows the related parameters, symbols, and units. Supplement 1 provides a detailed theoretic description (supplemental materials are available at http://jnm.snmjournals.org) (17).

**TABLE 2.** Parameters, Symbols, and Units Used in Study

Symbol	Description	Units
*C_i_*	Drug concentration in compartment	kBq/mL
*f_i_*	Drug fraction free from nonspecific binding	None
*k*_off_	Reverse rate constant	s^−1^
*k*_on_	Forward rate constant	mL · s^−1^ · kBq^−1^
*B*_max_	Maximum drug-specific binding concentration	kBq/mL
BP	Binding potential	None

### Acquisition and Reconstruction Protocols

All participants underwent ^123^I-FP-CIT SPECT (triple-head SPECT system GCA-9300R; Canon Medical), brain MRI (Titan 3-T MRI; Canon Medical), and CT imaging (SOMATOM Definition AS_mCT; Siemens). ^123^I-FP-CIT (167 MBq) was administered intravenously, with SPECT performed after 3 h to ensure radioligand equilibrium (18). Takahashi et al. provided a detailed description of the data acquisition and image reconstruction process (Supplement 2) (14).

### SBR Image Generation and Summed Voxel-Based SBR Measurement

**FIGURE 3.**
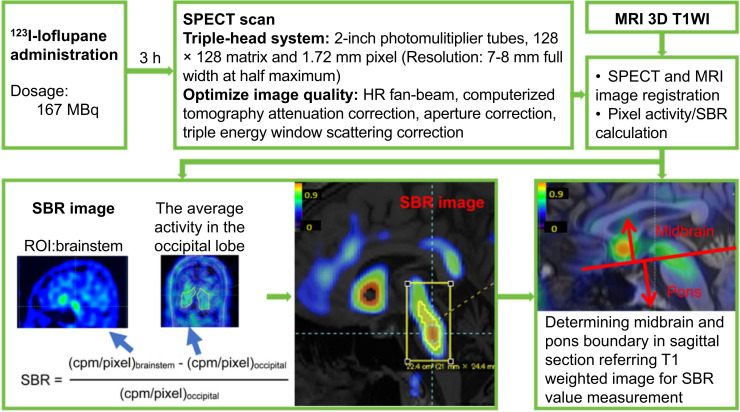
Experimental design flow for measuring summed voxel-based SBR in brainstem. Flowchart illustrates process of SBR image generation. Use of triple-head SPECT system and accurate image fusion with 3-T MRI system ensures reliable quantitative evaluation. cpm = count per minute. HR = high-resolution; T1WI = T1-weighted image.

The occipital lobe was selected as the reference region because of its low SERT distribution (12) and minimal pathologic involvement in MSA (12,19). 3D ROI was manually defined, and data for SBR calculation within this 3D ROI were automatically extracted using Mirada DBx version 1.1.1 software. The brainstem 3D ROI was selected using a threshold method with a lower threshold (SBR, 0.08) at 5% of the mean maximum SBR (1.71 ± 0.28) from the first 9 HCs to prevent individual measurement instability. The anatomic boundary between the midbrain and pons in the sagittal plane was accurately identified as described by Oba et al. referring to the registered MRI (20). The total SBRs within each manually defined 3D ROI for the midbrain and pons were then calculated (Fig. 3).

The brainstem’s SERT distribution is irregular, unlike striatal dopamine transporter SPECT uptake, which has a fixed shape and a relatively uniform distribution (21). Therefore, we assessed the summed voxel-based SBR by summing the SBRs of all voxels within the brainstem 3D ROI rather than using average SBR. Supplement 3 describes detailed procedures for generating SBR imaging and quantifying the summed voxel-based SBRs.

We conducted a phantom experiment using a ^123^I-filled brain torso phantom to validate the accuracy and robustness of our manual ROI-based total SBR quantification approach and assess potential effects of ROI size and system geometry (Supplement 4).

### Neuropathologic Assessment

Patients’ families gave written informed consent before autopsies following the guidelines of the ethical research committees of Aichi Medical University. We included 5 neurologically HCs and 5 representative autopsied cases of MSA-P, comprising striatonigral degeneration and α-synuclein-immunopositive glial cytoplasmic inclusions (22). The brains and spinal cords were fixed in 20% formalin for at least 2 wk, followed by paraffin embedding. Initially, the brainstem was cut at the valley between the superior and inferior colliculi of the midbrain, and 5-mm-thick slices were created from there toward the medulla oblongata. We prepared 6.5-μm-thick sections for immunohistochemical analysis. Anti-α-synuclein (polyclonal rabbit, 1:1,000; Sigma-Aldrich) was used as the primary antibody, together with antiphosphorylated α-synuclein (pSyn#64; monoclonal mouse, 1:1,000; Wako Pure Chemical Industries) and anti-SERT (1:500; monoclonal mouse; Millipore) antibodies. A standard avidin/biotin technique was used, and diaminobenzidine was used as the chromogen.

### Quantitative Assessment of SERT Expression in Autopsied Brains

**FIGURE 4.**
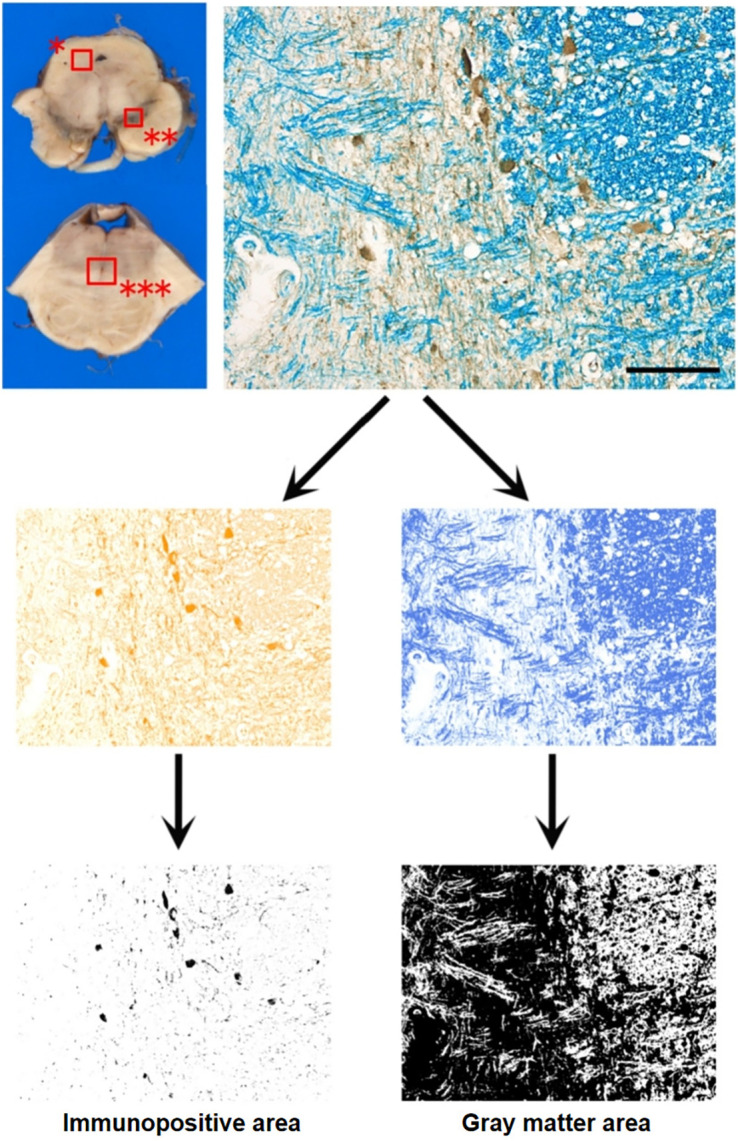
Quantitative analysis of SERT density. We investigated substantia nigra (**) of midbrain and raphe nucleus (***) of pons. Anti-SERT immunohistochemistry was combined with myelin staining using Luxol fast blue. Immunopositive area (stained with 3,3′-diaminobenzidine) and gray matter (Luxol fast blue–negative) were automatically detected using color deconvolution plugin in Fiji/ImageJ. SERT density was calculated as (immunopositive area/gray matter area). Scale bar indicates 50 μm.

We prepared midbrain sections at the valley level between the superior and inferior colliculi and midpons at the middle cerebellar peduncle level for representative MSA-P cases. Specimens were immunolabeled with an anti-SERT antibody and then myelin-stained with Luxol fast blue. Combining immunohistochemistry and Luxol fast blue counterstaining enabled us to compute the area of SERT immunostaining/gray matter. A charge-coupled device camera attached to the microscope under the same gain and light settings was used to acquire specimen photomicrographs at ×400 magnification. The color deconvolution plugin of Fiji/ImageJ was used to automatically identify SERT-immunopositive and Luxol fast blue–negative pixels (Fig. 4).

### Statistical Analysis

R (version 4.4.1; R Foundation for Statistical Computing) and RStudio (version 2023.06.1 + 524) were used for statistical analyses. The Mann–Whitney *U* test was conducted to perform comparisons between groups. Statistical significance was set at a *P* value of less than 0.05 for all analyses.

### ^123^I-FP-CIT SPECT Study

We compared summed voxel-based SBRs of patients with MSA-P and HCs across the brainstem, midbrain, and pons. Further, we conducted receiver operating characteristic curve analyses for the midbrain-to-pons ratio in MSA-P versus HCs.

### SERT Immunohistochemistry

SERT density in the substantia nigra and pontine raphe was assessed, and the ratio between them was calculated. A comparative analysis was conducted between 5 MSA-P cases and 5 HCs.

## RESULTS

### Participant Characteristics

The MSA-P group was younger than HCs, whereas the sex distribution was similar between groups (Table 1). To account for the potential confounding effect of age on SBRs, group comparisons were reanalyzed using analysis of covariance, with age as a covariate. In the pathologic study, the MSA-P and HC groups did not significantly differ in age or sex (*P* > 0.05) (Table 1).

### Effect of SSRIs on SBR Image

**FIGURE 5.**
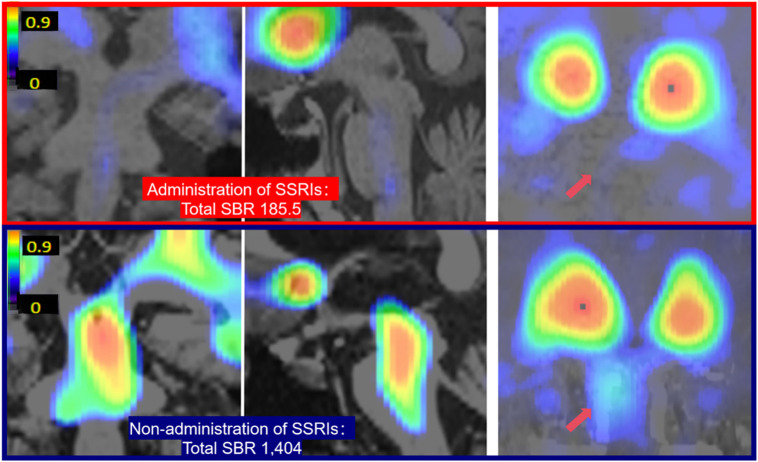
SBR images of brainstem uptake demonstrated notable reduction in patients taking SSRIs compared with those not on SSRIs. Arrows indicate brainstem uptake. Rainbow color scale represents SBR values.

In our study, 2 patients with MSA-P received SSRIs along with other medications. Both patients demonstrated significant reductions in summed voxel-based SBRs in the brainstem but not in the striatum (Fig. 5). This finding supports the idea that ^123^I-FP-CIT binds specifically to SERT in the brainstem. Intergroup analysis excluded these 2 cases. Supplement 5 shows the specific medications and dosages taken by these patients.

### Semiquantitative SBR Image Assessment

**FIGURE 6.**
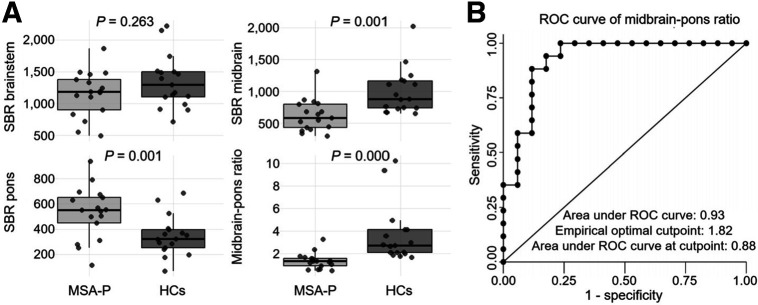
Midbrain-to-pons SBR ratio and diagnostic performance in MSA-P. (A) Summed voxel-based SBR was significantly reduced in midbrain and significantly increased in pons in MSA-P group. Consequently, midbrain-to-pons ratio was significantly lower in MSA-P group. (B) ROC curve of midbrain-to-pons ratio shows good performance. ROC = receiver operating characteristics.

The total SERT status in the brainstem was quantitatively assessed by summing the SBRs of all voxels within the 3D ROI for the brainstem. Figure 6A illustrates the comparisons of summed voxel-based SBR measurements for the brainstem, midbrain, pons, and brainstem-to-pons ratio between the 2 groups. No significant differences were observed in the brainstem between the MSA-P and HC groups. However, the MSA-P group demonstrated significantly lower SBRs in the midbrain and significantly higher values in the pons. Group differences remained after adjusting for age using analysis of covariance, although *P* values for most summed voxel-based SBRs were generally higher than in the unadjusted analysis, indicating that age is an important covariate affecting SBR measurements. Supplemental Table 1 presents detailed results before and after age adjustment. The midbrain-to-pons ratio was significantly lower in the MSA-P group. Further, the area under the curve of the midbrain-to-pons ratio was 0.93, with an empiric optimal cutoff of 1.82, using MSA-P as the positive reference (Fig. 6B). Furthermore, we conducted a correlation analysis between the summed voxel-based SBRs and clinical symptoms and have summarized the results in Supplemental Table 2.

We compared both total voxel-based and mean SBRs (Supplemental Table 1). Total SBR in the midbrain exhibited significant differences between groups, whereas the difference in mean SBR was not statistically significant. This discrepancy may be due to variations in 3D ROI volume defined by the same threshold (SBR, 0.08), which, under the same full width at half maximum, leads to greater partial-volume effects. Therefore, total SBR may provide a more robust estimate of overall SERT availability than does mean SBR. Supplemental Tables 3 and 4 provide detailed SBRs.

### SERT Expression in the Midbrain and Pons of Autopsied MSA-P

**FIGURE 7.**
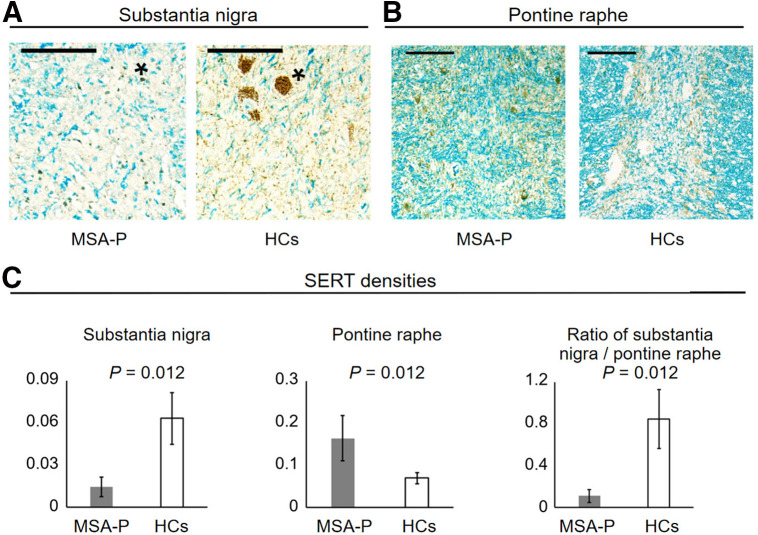
SERT expression in midbrain and pons of autopsied patients with MSA-P. (A) SERT-immunopositive neurites within substantia nigra were reduced in MSA-P case. (B) In contrast, pontine raphe nucleus of MSA-P case demonstrated increased SERT expression. Density of immunopositive neurites was increased. (C) Quantitative assessment revealed lower SERT density in substantia nigra of MSA-P cases than in HCs. MSA-P cases exhibited higher SERT density in pontine raphe nucleus than did HCs. Ratios of (substantia nigra/pontine raphe nucleus) for SERT density were lower in patients with MSA-P than in HCs. Scale bars indicate 100 μm (A) and 50 μm (B). A and B show anti-SERT immunohistochemistry. Asterisks indicate neuromelanin granules.

SERT immunopositivity is prominent within the neurites in the substantia nigra and pontine raphe nucleus in normal settings. SERT-immunopositive neurites within the substantia nigra were severely depleted in MSA-P (Fig. 7A). Conversely, the densities of SERT-immunopositive neurites within the pontine raphe nucleus were higher in patients with MSA-P (Fig. 7B). Quantitative analyses revealed significantly lower SERT densities in the substantia nigra and higher densities in the pontine raphe nucleus in patients with MSA-P than in HCs. The ratio of SERT density in the substantia nigra to that in the pontine raphe was significantly lower in the MSA-P group (Fig. 7C).

## DISCUSSION

We conducted a refined semiquantitative SERT assessment in the brainstem using a triple-head SPECT system to perform ^123^I-FP-CIT SPECT using SBR images based on the 3-compartment model and correlated the results with pathologic findings.

This study presented 3 key findings. First, SBR images revealed that midbrain SERT density was significantly lower and pons SERT density was significantly higher in the MSA-P group than in the HC group, consistent with our previous findings (14). The midbrain-to-pons ratio of the SBR exhibited an excellent receiver operating characteristic curve. Second, SERT-immunopositive neurites within the substantia nigra were severely depleted in MSA-P, whereas those in the pontine raphe nucleus increased. Quantitative assessments confirmed significantly lower and significantly higher SERT immunopositivity in the substantia nigra and pontine raphe nucleus, respectively, in MSA-P group than in HCs, aligning with ^123^I-FP-CIT SPECT results. Third, we assessed ^123^I-FP-CIT SPECT images of 2 patients with MSA-P taking SSRIs, and both demonstrated significantly lower brainstem SBRs than patients with MSA-P not taking SSRIs and HCs. This is consistent with our previous results, stating that ^123^I-FP-CIT uptake in the midbrain primarily reflects specific SERT binding (12,13).

We used a triple-head SPECT system, achieving a spatial resolution of approximately 7 mm in full width at half maximum, exceeding the 12 mm of dual-head systems. Considering the broader distribution range observed in the SBR images, we applied a lower threshold of SBR of 0.08 in the brainstem to calculate the summed voxel-based SBR across the entire brainstem. This threshold was selected to mitigate background noise in SBR images and was uniformly applied across all patient images. To ensure accuracy, 3D ROI boundaries were manually adjusted with reference to the T1-weighted MRI. This adjustment accounted for uptake extending beyond the anatomic structures because of resolution limitations. This method differs from software-automated analyses of brain morphology, including the statistical parametric mapping used in previous studies (23–25). Moreover, in SBR imaging, accurate boundary identification between the midbrain and striatum requires case-by-case manual judgment because of the varied distribution of these boundaries. Conversely, statistical parametric mapping analysis may introduce errors in SPECT image standardization. We aimed to achieve the most accurate SERT density quantification by measuring the summed voxel-based SBR in the brainstem. Chou et al. (26) investigated brainstem SERT in patients with MSA using [^11^C]3-amino-4-(2-dimethylaminomethylphenylsulfanyl)-benzonitrile PET and revealed increased binding in the dorsal raphe. However, they included a mix of MSA-P and MSA–cerebellar subtypes, using superior supratentorial white matter as the reference region. Suwijn et al. (27) used a SPECT system with a resolution of 6.5 mm in full width at half maximum after injection of ^123^I-β-CIT to acquire images after 24 h. They compared midbrain SERT-to-striatal dopamine transporter ratios, providing further insights into our study.

Decreases and increases in SERT can be interpreted as an adaptive response to serotonergic neurodegeneration to maximize the functionality of the remaining serotonergic neurons. SERT level increments are a compensatory mechanism in response to neurodegeneration. By increasing serotonin reuptake, synaptic serotonin concentration is modulated to prevent excessive neuronal stimulation. This increase may also elevate presynaptic serotonin concentration, thereby improving neurotransmission efficiency. Conversely, reduced SERT in the MSA-P group may reflect neuron degeneration and a compensatory phenomenon against decreased serotonergic neurons because a decrease in SERT indicates serotonin reuptake reduction, potentially leading to increased serotonin concentrations in the synaptic cleft. Such transporter level changes are analogous to observations in Parkinson disease, in which dopaminergic neuronal loss compensatingly reduces dopamine transporter levels to increase dopamine concentrations in the synaptic cleft (28). However, unlike dopamine transporter, SERT is expressed in neuronal cells, dendrites, and synapses, thereby complicating the understanding. Prospective changes in SERT in the midbrain and pons and their association with cerebrospinal fluid 5-hydroxyindoleacetic acid should be further investigated to fully understand the significance of these changes. This information could deepen our understanding of SERT dynamics in MSA-P and their correlation with clinical symptoms and disease progression, ultimately helping the development of more effective diagnostic and therapeutic strategies. Quantitative assessment revealed a wide range of total SBRs within the HC group, consistent with a previous report (29). Such variability may indicate potential compensatory mechanisms in the SERT system.

However, this study had some limitations. First, the low positive predictive value of the clinical diagnosis for MSA, along with the rarity of MSA, may have caused the misclassification or inclusion of other diseases in the MSA-P group (30). Second, our study involved manual 3D ROI determination; however, the upper midbrain may have been affected by overlap in uptake from the thalamus and striatum, as well as the potential presence of small amounts of dopamine transporter and other monoamines in the brainstem, which may have affected SERT evaluation. Third, we conducted an analysis of covariance to adjust for age as a covariate despite the age differences between the 2 groups. The group differences remained consistent after adjustment, reinforcing the robustness of our results and indicating that the observed differences are not affected by age-related factors. Fourth, direct comparison of absolute total SBRs between different studies may be limited by differences in spatial resolution and voxel size across γ-camera systems. The phantom experiments do not fully verify the theoretic basis of our approach; however, they provide partial support for its stability and reproducibility within the experimental settings. Further, the longer disease duration in the autopsy cohort compared with the imaging cohort reflects the inclusion of only deceased patients for postmortem analysis and represents an inherent study limitation.

## CONCLUSION

Our quantitative ^123^I-FP-CIT SPECT analysis revealed decreased midbrain and relatively increased pontine SERT density in MSA-P, consistent with pathologic SERT staining. These results indicate compensatory or reactive changes. The midbrain-to-pons SBR ratio demonstrated high diagnostic performance. The reduced uptake in SSRI-treated cases supports the SERT specificity of radioligand binding in the brainstem.

## DISCLOSURE

This study was supported by a Grant-in-Aid for Scientific Research (C) (grant 21K07470) and a Health and Labor Sciences Research Grant from the Ministry of Health, Labor, and Welfare, Japan (grant JPMH20FC1041). No other potential conflict of interest relevant to this article was reported.

## Notes

KEY POINTS **QUESTION:** Is there an abnormality observed in the serotonergic system in the brainstem of patients with MSA-P? **PERTINENT FINDINGS:** Compared with pathologic immunohistochemistry results, quantitative assessment of SERT density in the brainstem using ^123^I-FP-CIT SPECT revealed consistent findings of reduced SERT density in the midbrain and increased SERT density in the pons of patients with MSA-P. **IMPLICATIONS FOR PATIENT CARE:** These findings indicate that characteristic abnormalities may be observed in the serotonergic system in the brainstem of patients with MSA-P. This could provide a potential avenue for early diagnosis and disease monitoring. SPECT could be used to assess changes in SERT density and potentially improve management and treatment strategies for patients with MSA-P in clinical practice.
